# *Alu*-repeat polymorphism in the tissue plasminogen activator (*t-PA*) gene, seminal t-PA concentration, and male fertility impairment: A case-control study

**DOI:** 10.18502/ijrm.v13i8.7496

**Published:** 2020-08-19

**Authors:** Lubna Hamid Tahtamouni, Mahmoud Nael Hamdan, Zainab Ali Al-Mazaydeh, Randa Mahmoud Bawadi, Majdoleen Sobhi Rammaha, Ahmad Mohammad Zghoul, Mamoun Ahmad Ahram, Salem Refat Yasin

**Affiliations:** ^1^Department of Biology and Biotechnology, Faculty of Science, the Hashemite University, Zarqa, Jordan.; ^2^Department of Biochemistry and Molecular Biology, College of Natural Sciences, Colorado State University, Fort Collins, Colorado, USA.; ^3^Department of Physiology and Biochemistry, School of Medicine, the University of Jordan, Amman, Jordan.

**Keywords:** Alu element, Male infertility, Semen, Spermatogenesis, t-PA.

## Abstract

**Background:**

Tissue plasminogen activator (t-PA) is a protein involved in the fibrinolytic system that catalyzes the conversion of plasminogen into the active plasmin. The activity of t-PA is controlled by plasminogen activator inhibitor-1. t-PA has crucial functions during spermatogenesis. One polymorphism was reported for *t-PA* gene, either the presence of a 300-bp *Alu*-repeat (*Alu* ${}^{+}$) or its absence (*Alu* ${}^{-}$).

**Objective:**

The current work aimed at studying the association between *Alu* polymorphism in the *t-PA* gene and male infertility.

**Materials and Methods:**

Using polymerase chain reaction on genomic DNA isolated from the blood of 79 participants, a region polymorphic for *Alu* element insertion in *t-PA* gene was amplified. In addition, total t-PA concentration, plasminogen activator inhibitor-1 /t-PA complex concentration, and t-PA activity in seminal plasma were measured by enzyme-linked immunosorbent assay.

**Results:**

The results indicate that the percentage of infertile participants (n = 50) who were homozygous for *t-PA* *Alu* insertion (*Alu${}^{+/+}$*), heterozygous *Alu${}^{+/-}$* or homozygous for *t-PA* *Alu* deletion (*Alu${}^{-/-}$*) did not change significantly (p = 0.43, 0.81, and 0.85, respectively) when compared with the control participants (n = 29). On the other hand, a significant decrease (p = 0.0001) of t-PA total concentration in seminal plasma was observed in the infertile group in comparison with the control group. However, the results indicate that there is no association between the *t-PA* *Alu* different genotypes and the total t-PA seminal concentration in the infertile group when compared to the control group (p = 0.63).

**Conclusion:**

Data obtained from the current study does not support an association between *t-PA Alu* polymorphism and t-PA seminal concentration or male infertility.

## 1. Introduction

The male reproductive system consists of several organs that work together to produce sperm and other components of the semen (1). The seminiferous tubules are lined with epithelial cells that contain spermatogonial stem cells, which develop into healthy sperm, and Sertoli cells (2). In addition, the testes contain Leydig cells that secrete testosterone (3).

The primary hormones involved in the male reproductive system are testosterone, luteinizing hormone, and follicle-stimulating hormone. Testosterone is responsible for the development of male characteristics and regulates the gene expression or activates signaling pathways in Sertoli cells that are required to maintain spermatogenesis. Luteinizing hormone stimulates the production of testosterone by Leydig cells, and follicle-stimulating hormone is necessary to induce Sertoli cells to secrete androgen binding-protein (4-8).

Infertility is defined as the inability of couples in reproductive age to achieve pregnancy after one year of unprotected intercourse. Worldwide, approximately 10-15% of couples are considered infertile. According to the World Health Organization (WHO), male factors are diagnosed in almost 50% of infertility cases, either solely (20%) or in combination with female factor (30-40%) (9-12).

Sertoli cells play critical roles during spermatogenesis by providing optimal environment for germ cell development as well phagocytosing residual bodies shed by developing germ cells (13).

In addition, Sertoli cells are responsible for secreting tissue plasminogen activator (t-PA) (14-15). t-PA is a protein involved in the fibrinolytic system that converts plasminogen, an inactive proenzyme, into the active protease plasmin that dissolves fibrin clot. The extracellular proteolysis mediated by plasminogen activators (PA) is associated with many important biological processes such as tissue remodeling, tissue destruction, and cell migration, in addition to some reproductive events such as ovulation, luteolysis, and embryo implantation. The activity of t-PA is controlled by plasminogen activator inhibitor-1 (PAI-1), which regulates t-PA activity by binding to t-PA's active site, preventing the formation of plasmin (16-18).

In the human genome, *t-PA* is located at chromosome 8p12-p11.2 (19). Many studies on human population have indicated a 300-bp *Alu* repeat sequence insertion within intron 8 of the *t-PA* gene (20-23). One polymorphism was reported for this *Alu* repeat; either the presence of this repeat (Insertion/*Alu* ${}^{+}$) or its absence (Deletion/*Alu* ${}^{-}$) (21). The *Alu* polymorphism in the *t-PA *gene might play a role in the primary structure of the protein, and it may affect its secretion rate or plasma level (22). It was reported that there is an association between *Alu* polymorphism in the *t-PA *gene and t-PA plasma levels (23). However, the plasma level of t-PA is not only dependent on the secretion of t-PA but also on its rate and degree of complex-formation with PAI-1 (23). During spermatogenesis, t-PA has a role in the transport of preleptotene primary spermatocytes, through the blood-testis barrier, from the basal to the adluminal compartments of the seminiferous tubules. In addition, t-PA is involved during spermiation (14-15, 24-26). However, and as far as our knowledge is concerned, the association between *t-PA* *Alu* polymorphism and spermatogenesis has not been established. Thus, the current study aimed at studying the association between *Alu* polymorphism in the *t-PA* gene and male infertility.

## 2. Materials and Methods

### Study population

The current study was a case-control study conducted between May and December 2018. A total of 79 Jordanian males consulting gynecologists at different assisted reproduction centers in Amman, Jordan were recruited to participate in the current study. Patients were identified from patient's registry at the centers. All included participants signed a consent form.

### Sample collection

Blood samples were collected in EDTA blood collection tubes and seminal fluid samples were collected in sterile cups and were handled according to the WHO guidelines (27). Seminal fluid analysis (SFA) was performed according to the WHO guidelines (27).

### Alu polymorphism

Genomic DNA was extracted from blood samples of control and infertile men using a commercial genomic DNA purification kit (Wizard Genomic DNA Purification Kit, Promega, USA).

DNA amplification by polymerase chain reaction (PCR) in a thermal cycler (MyCycler, Bio-Rad, USA) was performed following these conditions: initial denaturation for 2 min at 96°C, followed by 35 cycles of denaturation for 30 sec at 96°C, annealing for 30 sec at 65°C and elongation for 30 sec at 96°C. The total volume of each reaction was 30 µl containing 1 µl of each forward (GTAACCATTTAGTCCTCAGCTGTTCTCCT) and reverse (CCATGTAAGAGTAGAAGGAGACTCAG- TCA) primers (28), 8 µl of nuclease-free water, 15 µl of the master mix (New England Biolabs, USA), and 5 µl of DNA sample.

PCR products were separated on 2% agarose gel electrophoresis containing 0.5 µg/ml ethidium bromide. Homozygote individuals carrying the *t-PA Alu* inserts are designated *Alu${}^{+/+}$*, heterozygotes as *Alu${}^{+/}{}^{-}$*, and homozygotes for the absence of the insert as *Alu${}^{-}{}^{/}{}^{-}$*.

### t-PA concentration and activity

Seminal total t-PA concentration was measured using the Human Total t-PA ELISA Kit (Assaypro LLC, USA). The absorbance on a microplate reader (Synergy HTX Multi-Mode Reader, USA) at a 450 nm was read immediately. On the other hand, seminal PAI-1/t-PA concentration was measured using the Human PAI-1/t-PA ELISA Kit (Assaypro LLC, USA). The activity of seminal t-PA was assayed using the Human t-PA Chromogenic Activity Kit (Assaypro LLC, USA). The absorbance was read at 405 nm for a zero-minute background reading and then was read every 1 hr on a microplate reader (Synergy HTX Multi-Mode Reader, USA) for 6 hr.

### Ethical consideration

The study was approved by the Institutional Review Board (IRB) of the Hashemite University, which conforms to the World Medical Association Declaration of Helsinki (code: KTB/16/11/1800801).

### Statistical analysis 

All genotypes and frequencies for the insertion or deletion of the recruited individuals were calculated according to the counting method. The observed genotypes and alleles frequencies were compared with those expected in order to verify the Hardy-Weinberg equilibrium. To determine the differences between the two means, the Student's *t* test was performed, while the difference between the two proportions was calculated using the two-proportion test. Factorial ANOVA for higher orders (2-way or 3-way) was used to test for interactive effects for multiple categorical independent variables. Statistical analysis was performed using the Statistica software, StatSoft Inc., Tulsa, OK, USA (version 10). *P*-value < 0.05 was considered statistically significant.

## 3. Results

Seminal fluid was collected from the 79 participants and analyzed. Twenty-nine samples were normal (29.8 ± 4.3 yr) according to the WHO criteria (27), while fifty samples were considered as abnormal (36.7 ± 6.7 yr; Table I).

**Figure 1:**
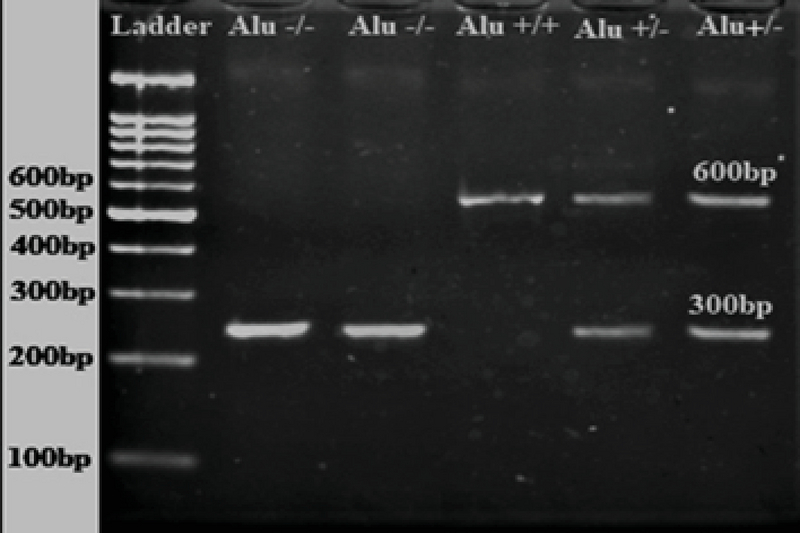
Representative amplified *Alu* segment of the *t-PA* gene showing insertion (*Alu${}^{+/+}$*) and/or deletion (*Alu* ${}^{-/-}$) of the segment. Lane 1:100 bp Ladder; Lanes 2 and 3: *Alu* deletion (*Alu${}^{-/-}$*) at 300 bp; Lane 4: *Alu* insertion (*Alu${}^{+/+}$*) at 600 bp; Lanes 5 and 6: *Alu* insertion and deletion (*Alu${}^{+/-}$*) at 300 bp and 600 bp.

The *Alu* element of the *t-PA* gene was successfully amplified from all genomic DNA samples and PCR products were examined using 2% w/v agarose gel electrophoresis (Figure 1). *Alu *genotypes and allelic frequency of *Alu* insertion/deletion of the *t-PA* gene in control and infertile participation are shown in Table II. The results indicated that the percentage of infertile participants who were homozygotes for *Alu${}^{+/+}$*was slightly decreased (66%) compared to the control group (75.9%); however, this decrease was insignificant (p = 0.43). On the other hand, the percentage of both *Alu${}^{-/-}$*homozygotes and heterozygotes *Alu${}^{+/-}$* were insignificantly increased in the infertile group (16%, and 18%, respectively) when compared to the control group (10.3% and 13.8%, respectively) (p = 0.81, and 0.85, respectively). The allelic frequency of the *Alu* ${}^{-}$ was decreased for the infertile group (0.74) when compared to the control group (0.81), though the decrease was insignificant (p = 0.48).

Total t-PA concentration, PAI-1/t-PA complex concentration, and t-PA activity in seminal plasma were assessed by ELISA assay for the control and infertile groups (Table III). The results show that the total seminal t-PA (free and complexed with PAI-1) was significantly lower in the infertile group in comparison to the control group (p = 0.0001, Table III). On the other hand, the concentration of seminal t-PA complexed with PAI-1 in the infertile group was higher than the control group; however, this increase was insignificant (p = 0.19). The decrease in concentration of total seminal t-PA and the increase in t-PA/PAI-1 reflected in reduced activity of t-PA in the infertile group, though this reduction was insignificant (p = 0.68, Table III).

In order to assess if there was an association between *Alu* polymorphism in the *t-PA* gene and the total t-PA concentration in seminal plasma, multifactorial ANOVA test was performed. The results shown in Table IV indicate that the total t-PA concentration in the three *t-PA* *Alu* genotypes of the infertile group was lower than those of the control group. However, the statistical analysis of the results indicate that there was no association between the different *t-PA* *Alu* genotypes and the total t-PA seminal concentration in the infertile group when compared to the control group (p = 0.63, Table IV).

**Table 1:** Seminal fluid analysis of the 79 Jordanian male participants


**Type of sample**	**n (%)**	**Sperm concentration ×10${}^{6}$/mL**	**Progressive motility**	**Total motility**	**Normal morphology**
**Control**	29 (36.7)	75.0 ± 27.2	33.7 ± 3.2	62.8 ± 9.0	5.3 ± 2.0
**Infertile**	50 (63.3)	30.4 ± 26.0	4.3 ± 5.3	31.5 ± 14.0	1.6 ± 0.8
Note: Data are expressed as Mean ± SD; n, sample size					

**Table 2:** *Alu* genotypes and allelic frequency of *t-PA *gene in control and infertile participants


**Genotype**	**Control group % (n)**	**Infertile group % (n)**	**P-value**	**95% Confidence interval (CI)**
**Alu${}^{+/-}$**	75.9% (22)	66% (33)	0.4327	-15 to 31.4
**Alu${}^{+/-}$**	10.3% (3)	16% (8)	0.8107	-49 to 41.6
**Alu${}^{+/+}$**	13.8% (4)	18% (9)	0.8514	-45.4 to 39.1
**Allele**	Control group (n = 29)	Infertile group (n = 50)	
**Alu${}^{-}$**	0.81	0.74	0.4789	-13.2 to 24
**Alu${}^{+}$**	0.19	0.26	0.4789	-13.2 to 24
Note: Data are expressed as percentage; *P*-value of infertile group compared to that of the control group (two-proportion test)				

**Table 3:** Total t-PA concentration and activity in seminal plasma of control and infertile groups


	**Control group n = 29**	**Infertile group n = 50**	**P-value**	**95% Confidence interval (CI)**
**Total t-PA concentration (ng/ml)**	28.6 ± 17.5	14.0 ± 7.8	0.0001	8.9 to 20.3
**PAI-1/t-PA concentration (ng/ml)**	1.6 ± 0.7	1.9 ± 1.1	0.1908	-0.75 to 0.15
**t-PA activity (IU/ml)**	72.5 ± 24.3	70.1 ± 25.7	0.6844	-9.3 to 14.1
Note: Data are expressed as Mean ± SD; P-value of the infertile group compared to that of the control group (the Student's *t* test)				

**Table 4:** Total t-PA concentration in seminal plasma for *t-PA Alu *genotypes in control and infertile groups


	**** ***Alu*** **${}^{-/-}$**		**** ***Alu*** **${}^{+/-}$**		**** ***Alu*** **${}^{+/+}$**		
	**Control 75.9%**	**Infertile 66%**	**Control 10.3%**	**Infertile 16%**	**Control 13.8%**	**Infertile 18%**	**P-value**
**Total t-PA **								
**concentration (ng/ml)**	28.10 ± 18.53	13.9 ± 8.7	25.70 ± 2.01	15.6 ± 5.9	33.44 ± 19.85	13.1 ± 5.6	0.6342
Note: Data are expressed as Mean ± SD; P-value of the infertile group (n = 50) compared to that of the control group (n = 29) (factorial ANOVA for higher orders)							

## 4. Discussion

In the current study, total t-PA concentration, PAI-1/t-PA complex concentration, and t-PA activity in seminal plasma for control and infertile groups were determined (Table III). The results show that the total seminal t-PA (free and complexed with PAI-1) was significantly lower in the infertile group in comparison with the control group (p = 0.0001, Table III). On the other hand, the concentration of seminal t-PA complexed with PAI-1 in the infertile group was higher than the control group; however, this increase was statistically insignificant (p = 0.19). The decrease in the concentration of the total seminal t-PA and the increase in the t-PA/PAI-1 were reflected in reduced activity of t-PA in the infertile group, although this reduction was insignificant (p = 0.68; Table III).

t-PA is a protein involved in the fibrinolytic system that converts the proenzyme plasminogen into the active protease plasmin that dissolves fibrin clot (17, 18). In addition, the extracellular proteolysis mediated by t-PA is associated with many important biological processes, such as tissue remodeling, tissue destruction, and cell migration. The activity of t-PA is controlled by PAI-1 (17, 18).

t-PA has been implicated in many diseases such as thrombotic disorders including strokes and myocardial infarction (29). In addition, it was found to be involved in the process of angiogenesis in cancer cells (30). Its concentration was reported to be increased in breast cancer and myocardial infarction patients. Furthermore, it has also been associated with polycystic ovary syndrome (PCOS) (29). Moreover, it was postulated that it has a role during spermatogenesis. Sertoli cells-secreted t-PA plays a role in the transport of preleptotene primary spermatocytes, through the blood-testis barrier, from the basal to the adluminal compartments of the seminiferous tubules. In addition, t-PA was also shown to play a role in the process of spermiation at stages IX-XII (14, 15, 24-26).

It was reported that the limiting factor of how much active t-PA is available in an organ is the capacity of the organ endothelium to increase its secretion of t-PA when required, that is, the major physiological regulator of local (organ level) fibrinolytic capacity is the secretion rate of t-PA (16, 18).

A single study reported the relation of t-PA concentration with male infertility. A higher concentration of t-PA was found in normal male compared with oligo/azoospermia patients. In addition, a higher number of immotile spermatozoa in the semen was associated with higher t-PA activities (31). The results presented in the current study is in accordance with their results in terms of concentration, but not activity (Table III). A possible explanation for the lower t-PA concentration could be due to the malfunction of the Sertoli cells, which it is the source of t-PA in the testis (14, 15, 24-26). However, testing this hypothesis is beyond the scope of this work.

*Alu* DNA fragment is a major driving force for evolution. The *Alu* element affects genes either negatively via inactivation of genes or positively by altering their function (32). The *Alu* (I/D) *t-PA* polymorphism occurs in intron 8 of the *t-PA* gene and consists of the presence (insertion; I or +) or absence (deletion; D or -) of *Alu* fragment. The three *t-PA* *Alu* genotypes are *Alu${}^{+/+}$*, *Alu${}^{-/-}$,* and the heterozygote genotype *Alu${}^{+/-}$* (21). A genetic polymorphism may affect the primary structure of a protein, but may also affect its synthetic rate or its plasma level if the protein involved is a secretory protein. However, several studies reported that *Alu* (I/D) polymorphism in *t-PA* locus gene does not affect the synthesis rate of t-PA (33, 34). Nevertheless, one study reported that homozygous carriers of *Alu* (I/I) t-PA polymorphism have an increased “in vivo” release rate of t-PA from vascular endothelial cells, even though this polymorphism is located in a non-coding area of the *t-PA* gene (22).

In the current study, the association between the total t-PA concentration and *t-PA Alu *genotypes in the normal and infertile participants was determined. The results indicate that there was no association between the different *t-PA* *Alu* genotypes and the total t-PA seminal concentration in the infertile group when compared to the control group (p = 0.63; Table IV). The small sample size of the current study could be the reason behind this insignificant association.

## 5. Conclusion

In conclusion, data obtained from the current study does not support the presence of an association between *t-PA Alu* polymorphism and t-PA seminal concentration or male infertility.

##  Conflict of Interest 

The authors declare that there is no conflict of interest.
